# Supplementary figures and images for: Photosensing and quorum sensing are integrated to control Pseudomonas aeruginosa collective behaviors
Source: PLoS Biol. 2019 Dec 12;17(12):e3000579. doi: 10.1371/journal.pbio.3000579 (PMC6932827; doi:10.1371/journal.pbio.3000579)

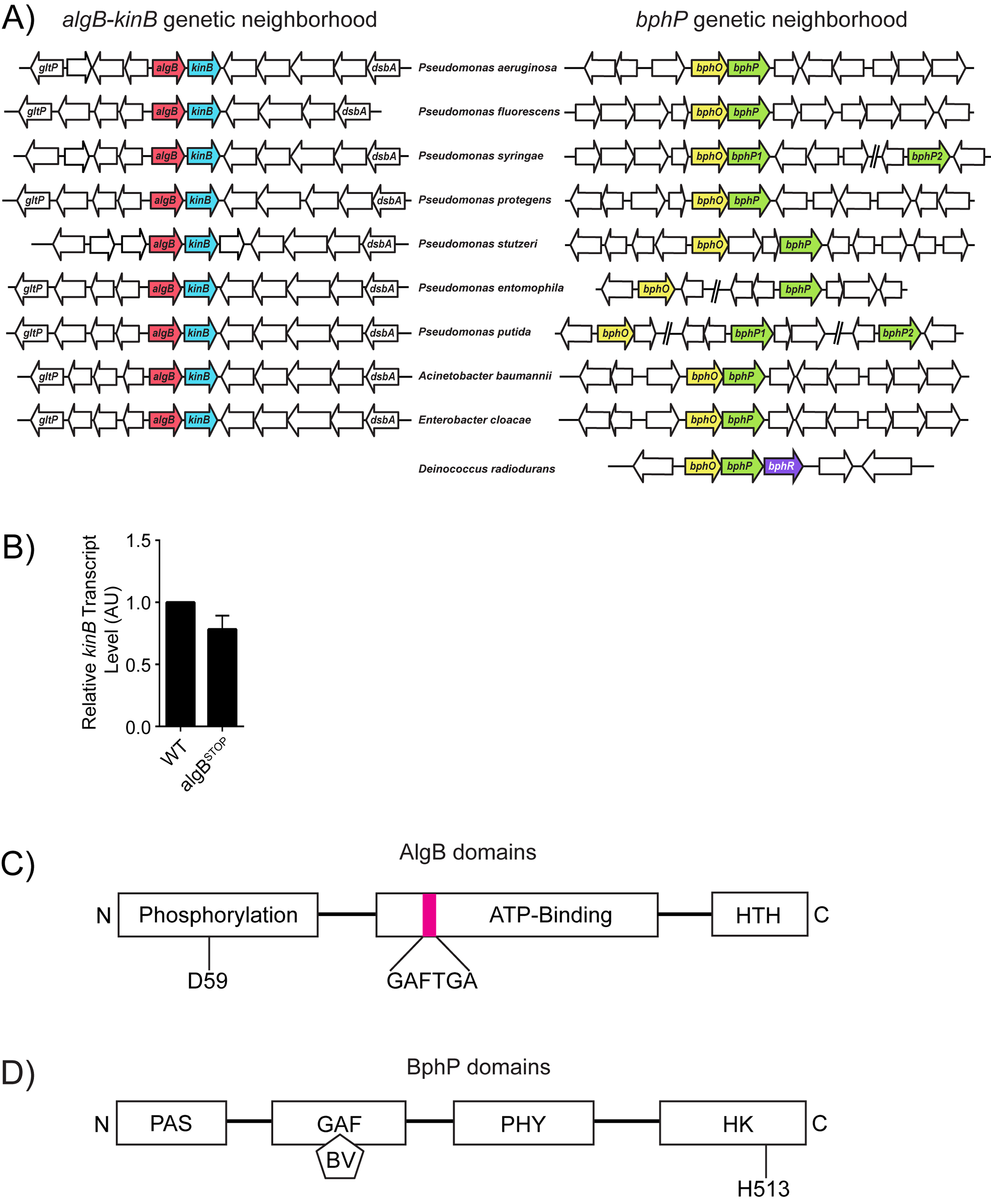

Supplement: S1 Fig — (A) The genes flanking kinB, algB, bphO, and bphP are diagrammed for the indicated genomes. The relative gene positions and orientations are accurate, but gene lengths are not to scale. (B) Relative expression of kinB measured by qRT-PCR in WT PA14 and the algBSTOP mutant grown planktonically to OD600 = 1.0. Data were normalized to 16S RNA levels, and the WT levels were set to 1.0. Error bars represent SEM for 3 biological replicates. (C) The domain architecture of the AlgB monomer is shown. Residue 59 is required for phosphorylation; the GAFTGA motif, indicated by the magenta shading, is required for interaction with σ54; and HTH refers to the helix-turn-helix DNA binding domain. Adapted from [31]. (D) Domain organization of the BphP monomer consisting of the PAS, GAF, PHY, and HK domains is shown. BV binds to the GAF domain, and residue H513 is required for autophosphorylation. Adapted from [17]. Data for panel B can be found in supplemental file S1 Data. AU, arbitrary unit; BV, biliverdin; GAF, cGMP-specific phosphodiesterases, adenylate cyclases, and FhlA; HK, histidine kinase; PAS, Per-Arnt-Sim; PHY, phytochrome; qRT-PCR, quantitative Reverse Transcriptase-Polymerase Chain Reaction; SEM, standard error of the mean. (TIF) [file pbio.3000579.s001.tif]

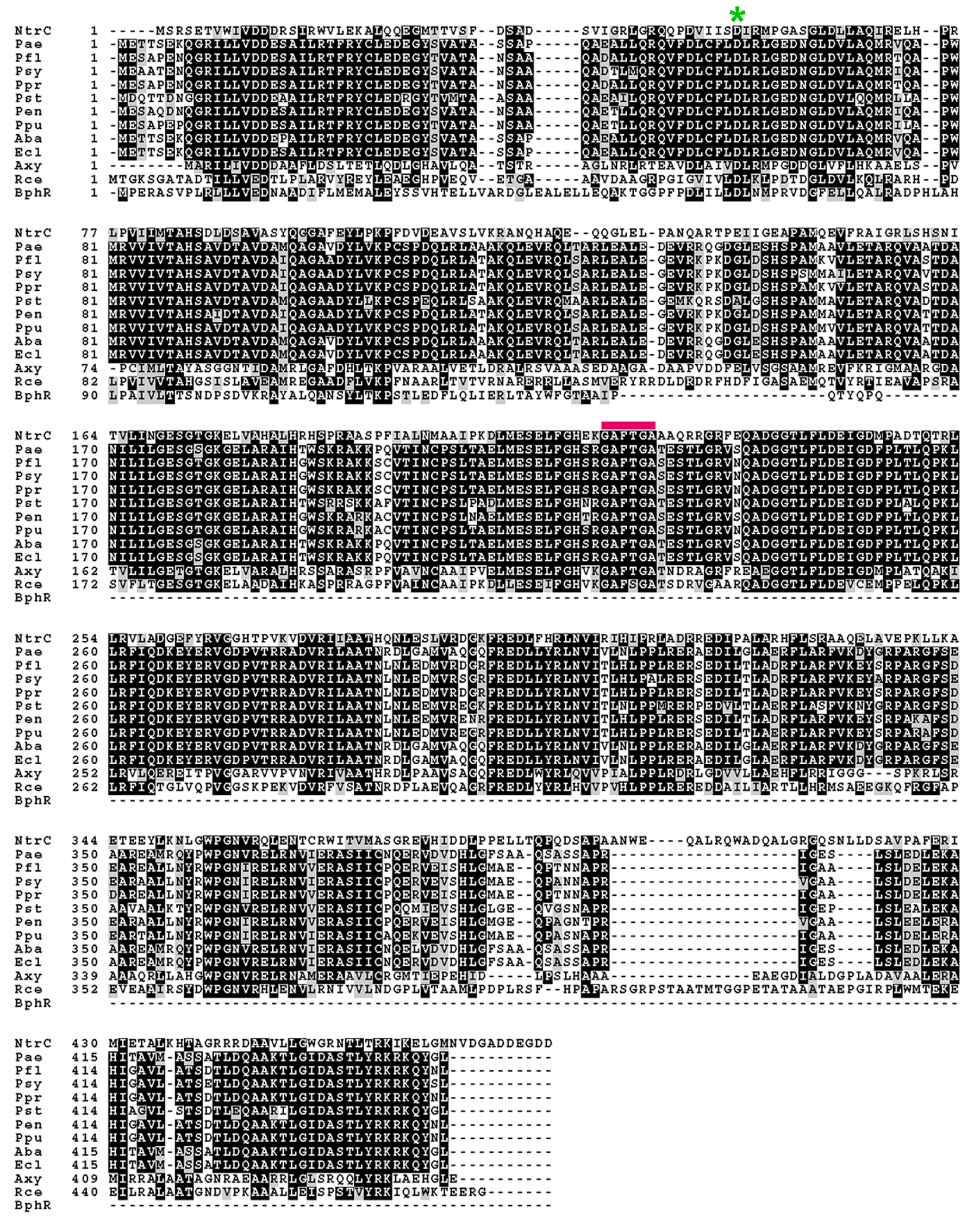

Supplement: S2 Fig — Primary sequence alignment of NtrC (first line) and AlgB (second line) from Pae and AlgB orthologs (third through twelfth lines) from Pfl, Psy, Ppr, Pst, Pen, Ppu, Aba, Ecl, Axy, Rce, and BphR (thirteenth line) from Deinococcus radiodurans. Highly conserved amino acids are highlighted in black. Residue 59 is shown by the green asterisk. The GAFTGA motif required for interaction with σ54 is indicated by the magenta line. Aba, Acinetobacter baumanii; Axy, Achromobacter xylosoxidans; Ecl, E. cloacae; Pae, P. aeruginosa; Pen, P. entomophila; Pfl, P. fluorescens; Ppr, P. protegens; Ppu, P. putida; Pst, P. stutzeri; Psy, P. syringae; Rce, R. centenum. (TIF) [file pbio.3000579.s002.tif]

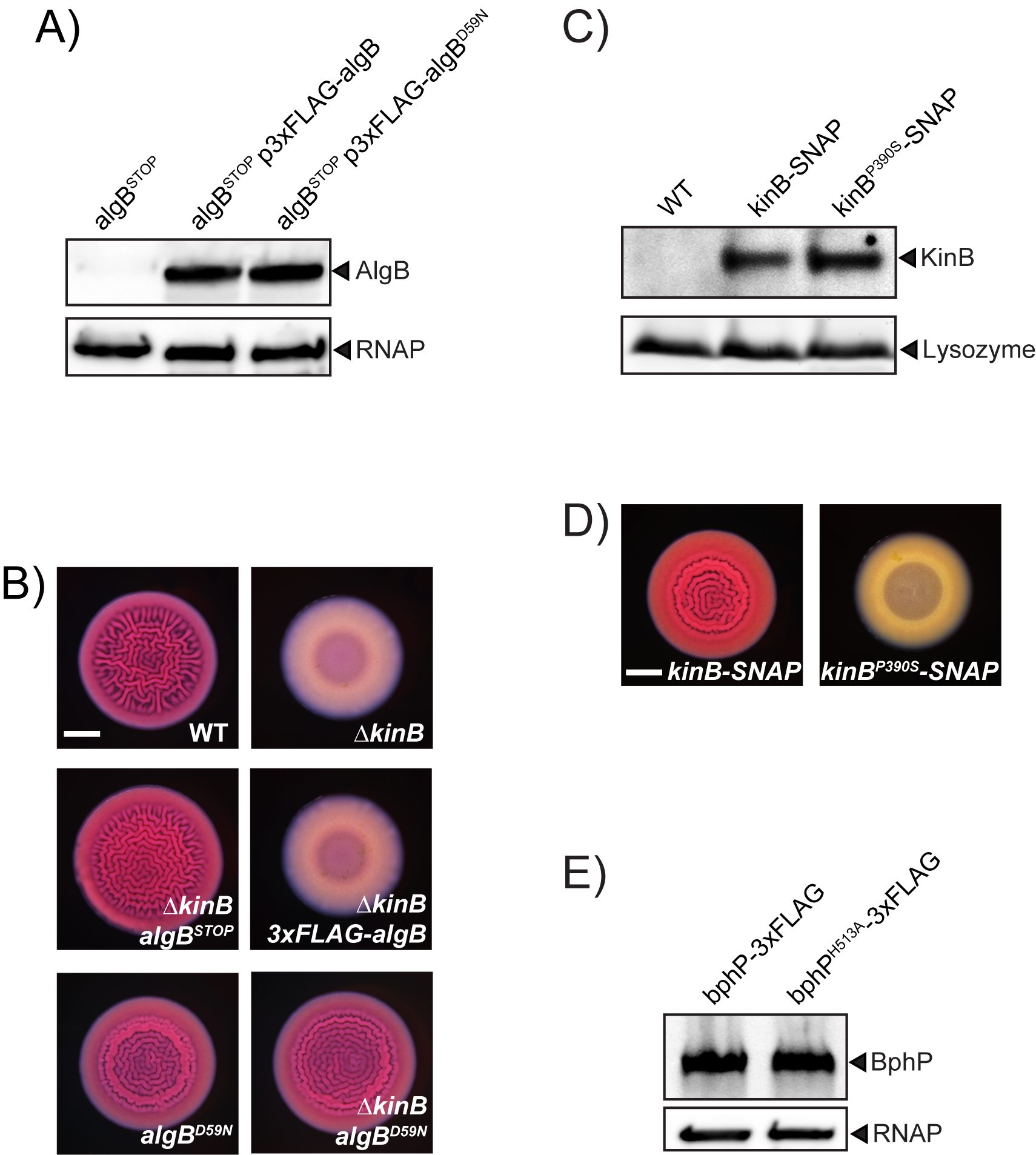

Supplement: S3 Fig — (A) Western blot analysis of whole cell lysates from the indicated strains, all of which have the algBSTOP allele at the native locus in the genome and carry an empty vector or 3xFLAG-algB or 3xFLAG-algBD59N on the pBBR1-MCS5 plasmid under the Plac promoter. The same cell lysates were probed for RNAP as the loading control. (B) Colony biofilm phenotypes of WT PA14 and the designated mutants. Scale bar is 2 mm. (C) SDS-PAGE analysis of whole cell lysates from the indicated strains. The gel was stained for SNAP using SNAP-Cell 647-SiR fluorescent substrate (New England Biolabs, Ipswich, MA, USA). Lysozyme was added as the loading control. (D) Colony biofilm phenotypes of the kinB-SNAP and kinBP390S-SNAP strains. Scale bar is 2 mm. (E) Western blot analysis of whole cell lysates from the indicated strains. The same cell lysates were probed for RNAP as the loading control. The original western blots showing the data for panels A, C, and E are available in supplemental file S2 Data. RNAP, RNA Polymerase; WT, wild type. (TIF) [file pbio.3000579.s003.tif]

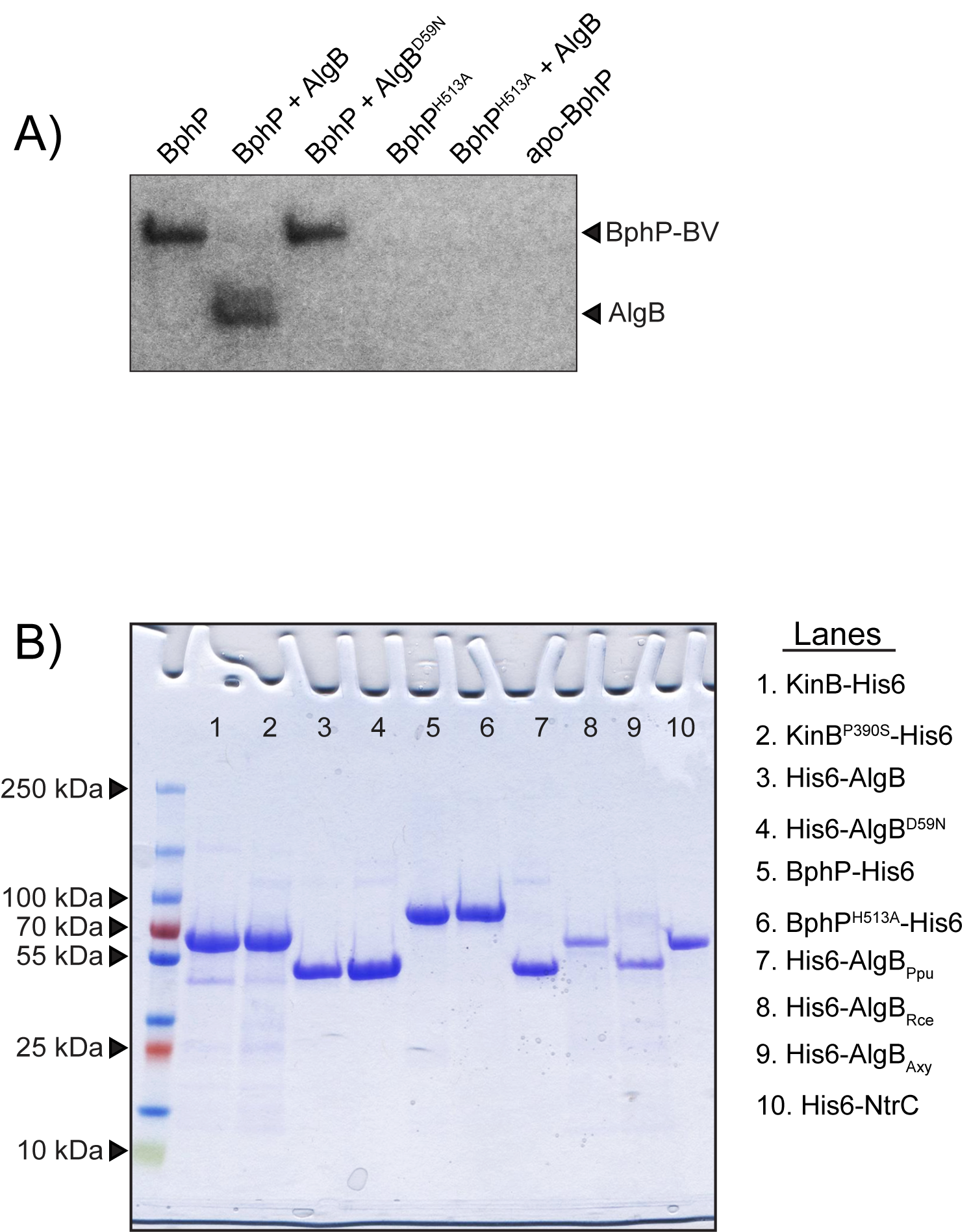

Supplement: S4 Fig — (A) Autophosphorylation of the BphP–BV complex was carried out for 30 min (leftmost lane), followed by addition of AlgB (second lane) or AlgBD59N (third lane) for an additional 30 min. The kinase-defective BphPH513A-BV complex was incubated with radiolabeled ATP for 30 min (fourth lane), followed by addition of AlgB (fifth lane) for an additional 30 min. The apo-BphP protein was incubated with radiolabeled ATP for 30 min (sixth lane). (B) SDS-PAGE gel stained with Coomassie brilliant blue showing the indicated purified proteins. Ten μL of a 20 μM stock of each protein was loaded. The original autoradiograph showing the data for panel A is available in the supplemental file S2 Data. BV, biliverdin. (TIF) [file pbio.3000579.s004.tif]

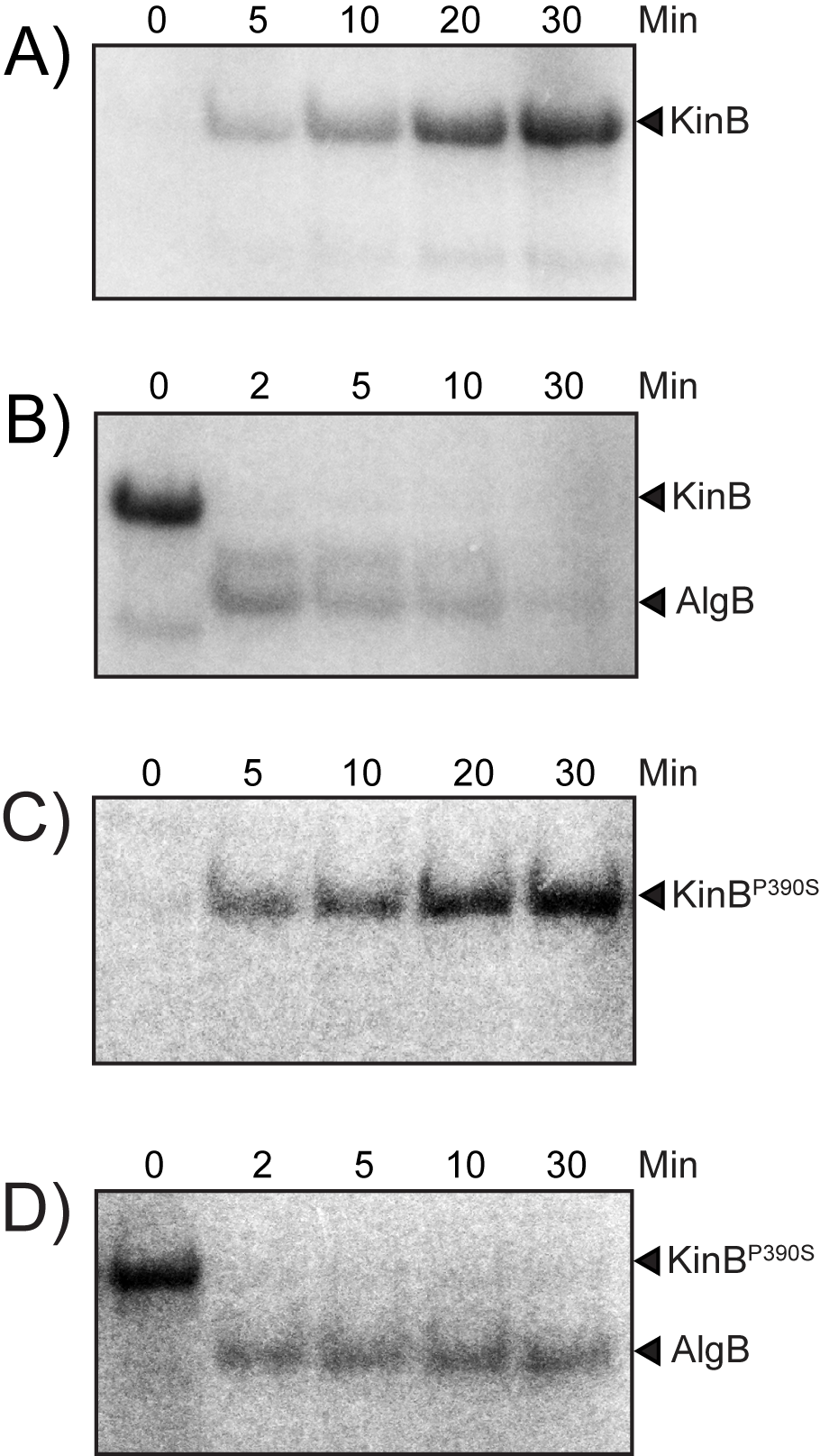

Supplement: S5 Fig — (A) Autophosphorylation of KinB was carried out for 30 min, and samples were removed at the indicated times. (B) An equimolar amount of AlgB was added to KinB that had been autophosphorylated for 30 min as in (A). Samples were taken at the indicated times. (C and D) As in A and B, respectively, but for the phosphatase-deficient protein KinBP390S. The original autoradiographs with the data for this figure are available in supplemental file S2 Data. (TIF) [file pbio.3000579.s005.tif]

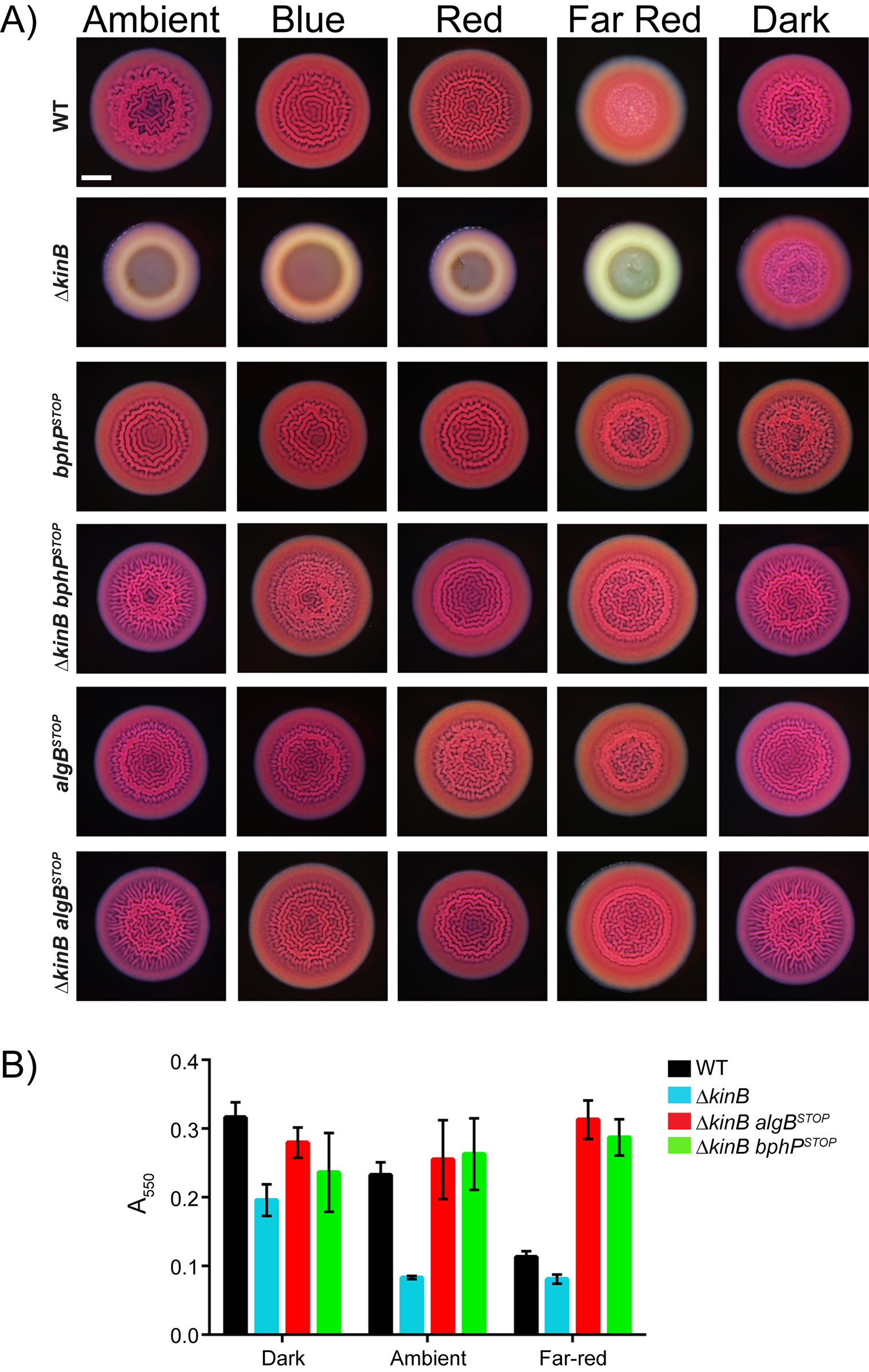

Supplement: S6 Fig — (A) Colony biofilm phenotypes are shown for WT PA14 and the designated mutants on Congo red agar medium after 72 h of growth under the indicated light conditions. Scale bar is 2 mm for all images. (B) SSA biofilm phenotypes assessed by crystal violet staining are shown for WT PA14 and the designated mutants after 72 h of growth under the indicated light conditions. Data can be found in supplemental file S1 Data. SSA, solid-surface–associated; WT, wild type. (TIF) [file pbio.3000579.s006.tif]

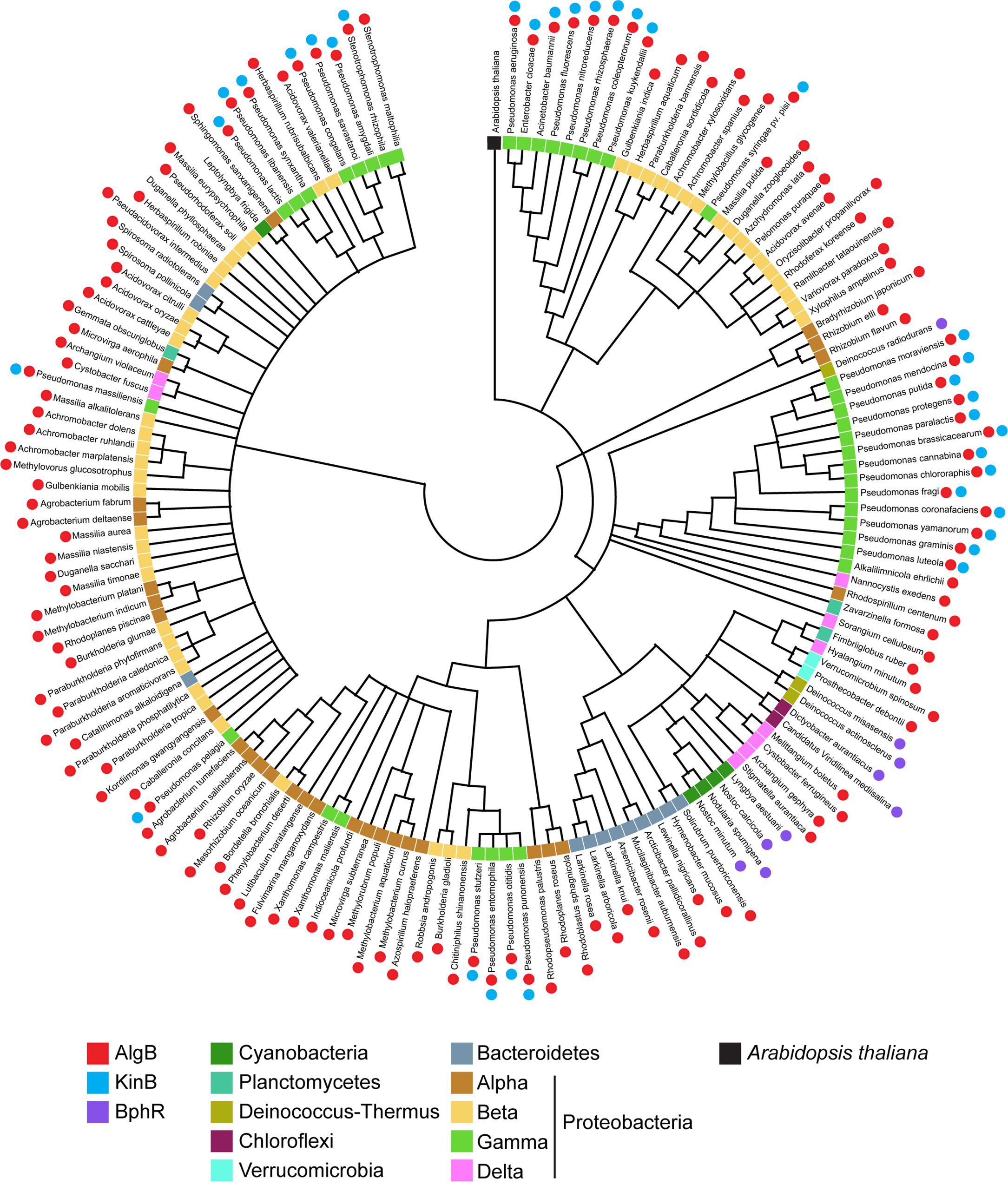

Supplement: S7 Fig — Enlarged maximum-likelihood–based phylogenetic tree for BphP from Fig 6A showing the 150 closest orthologs to P. aeruginosa BphP. Co-occurrences of AlgB and KinB are depicted using red and blue dots, respectively. The presence of BphR is shown by purple dots. The colored squares indicate the corresponding bacterial phyla. The black square indicates A. thaliana as the root of the tree. (TIF) [file pbio.3000579.s007.tif]

Fig 2C

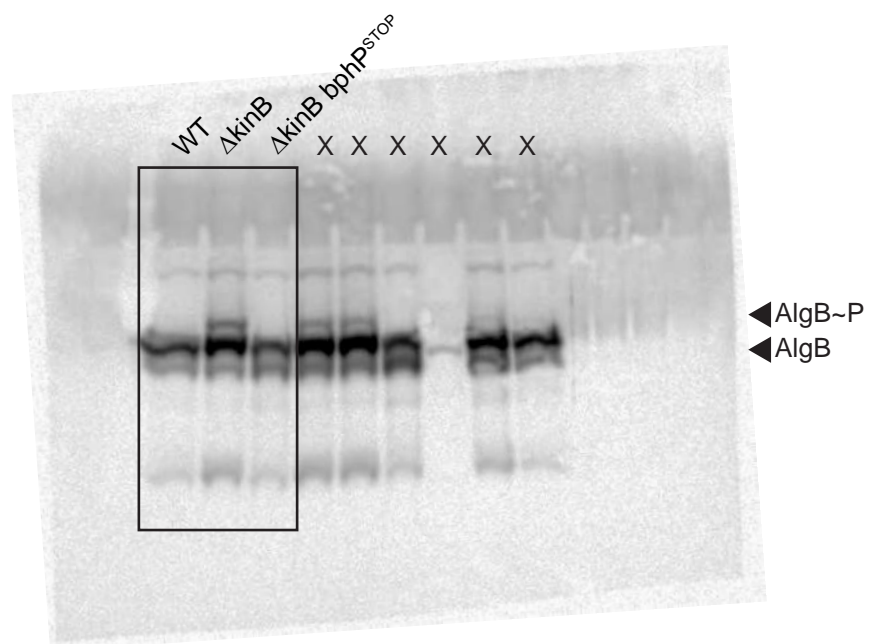

Fig 3E

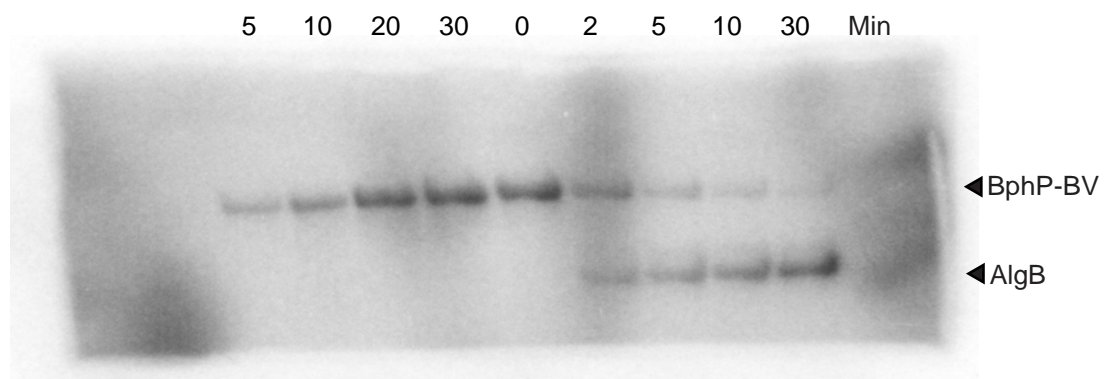

Fig 3F

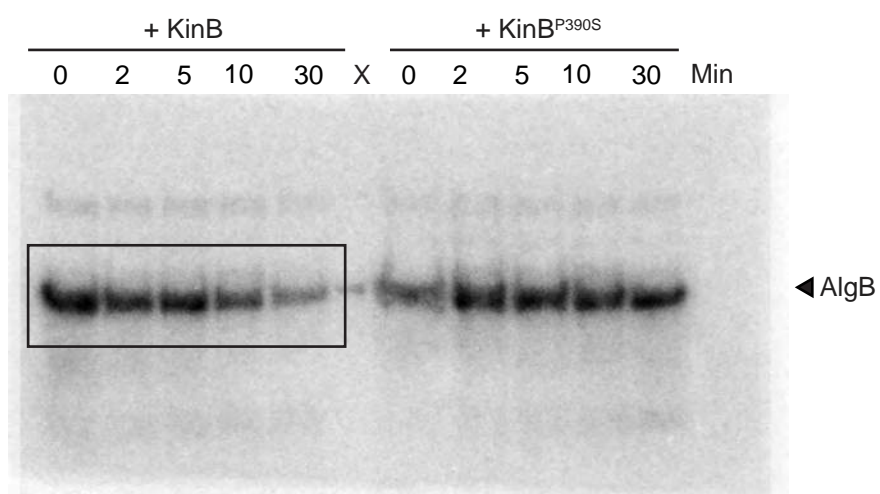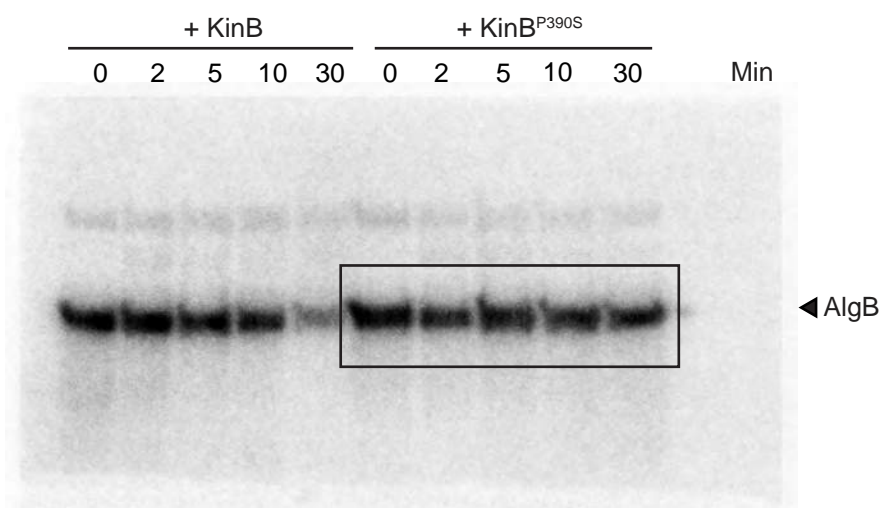

Fig 4B

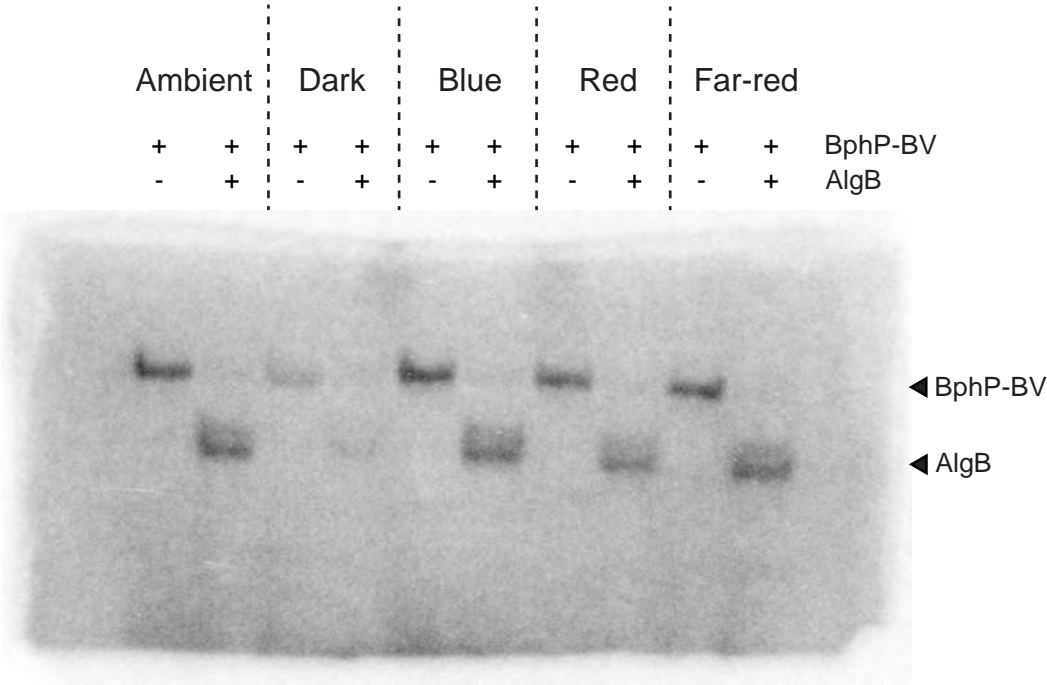

Fig 6B

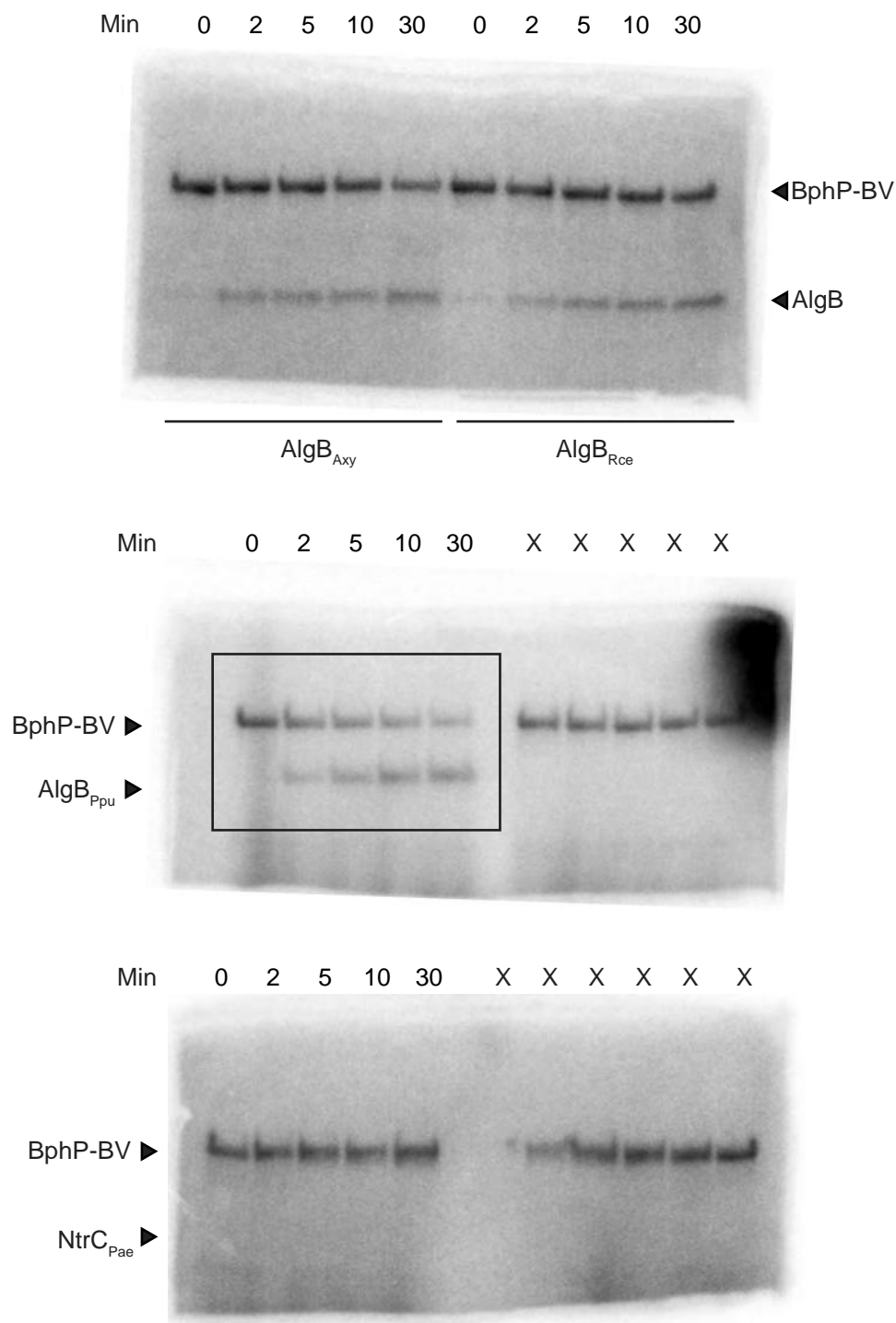

S3 Fig

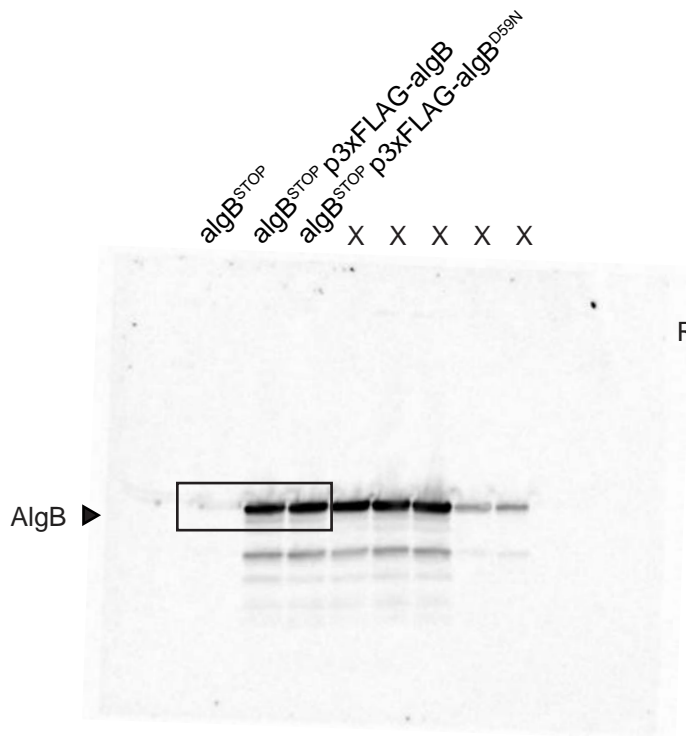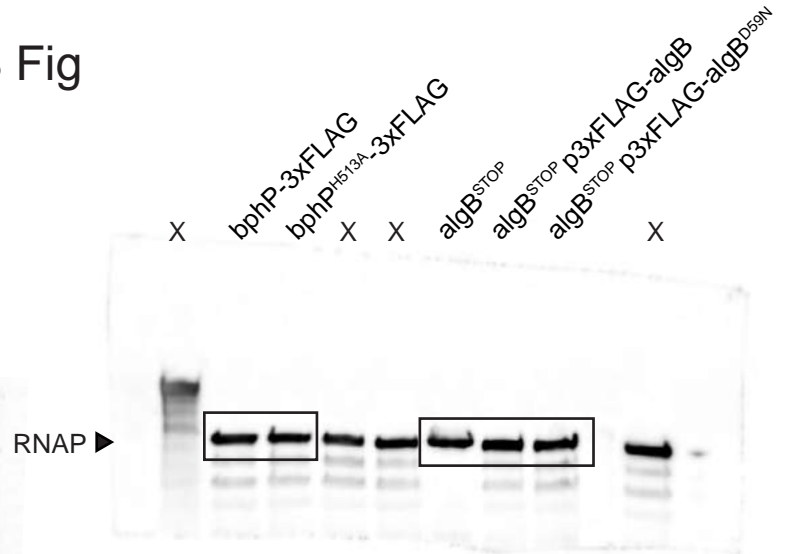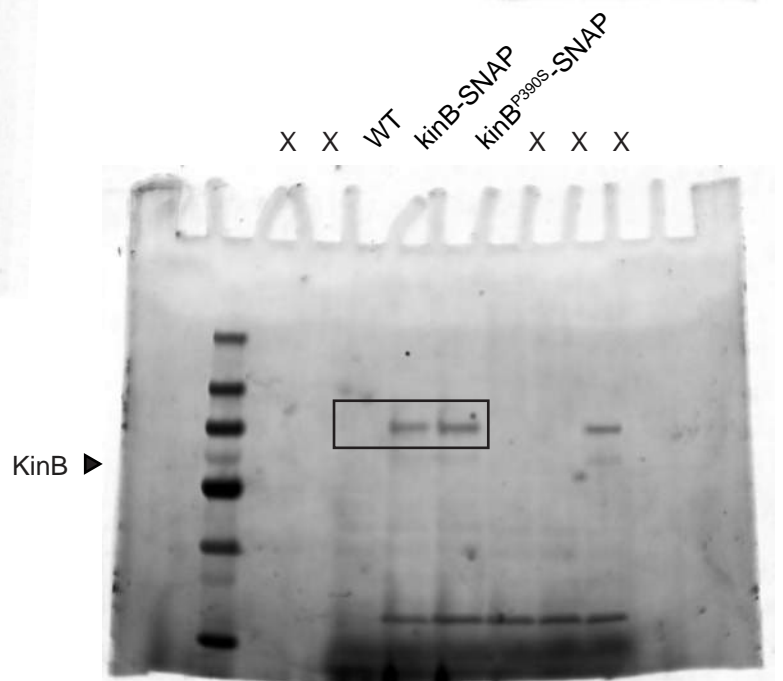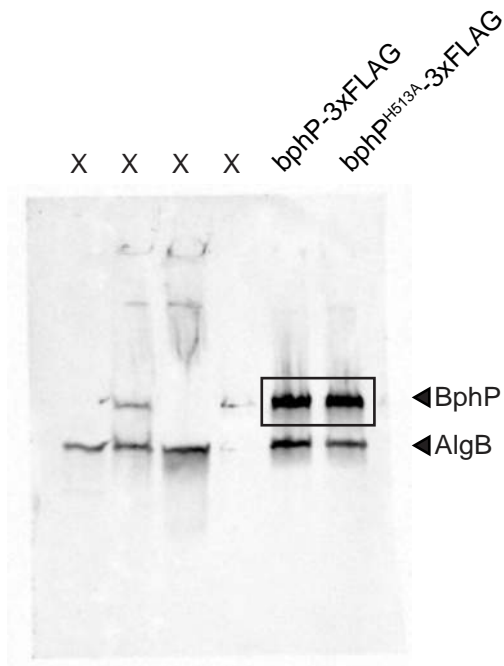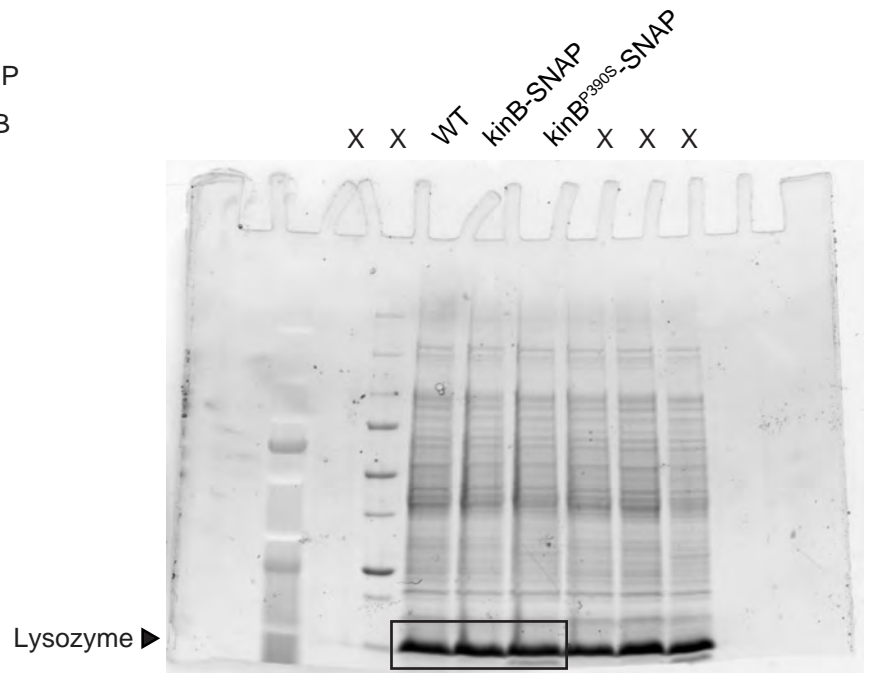

S4 Fig

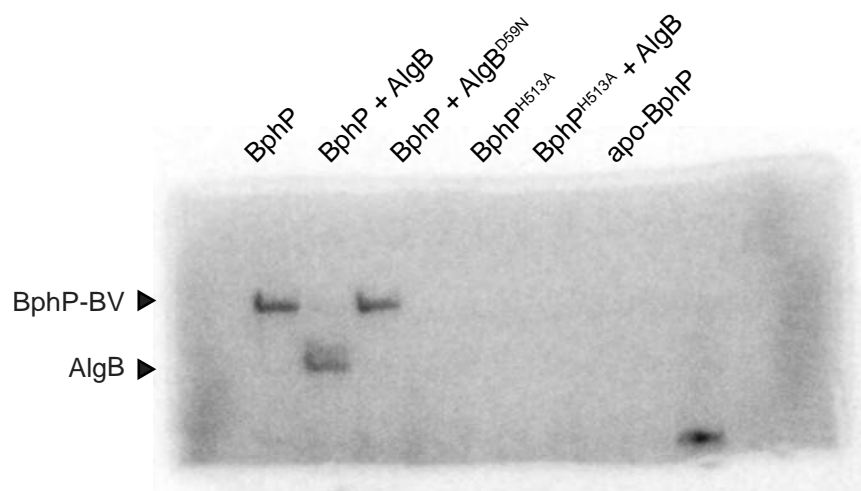

# S5 Fig

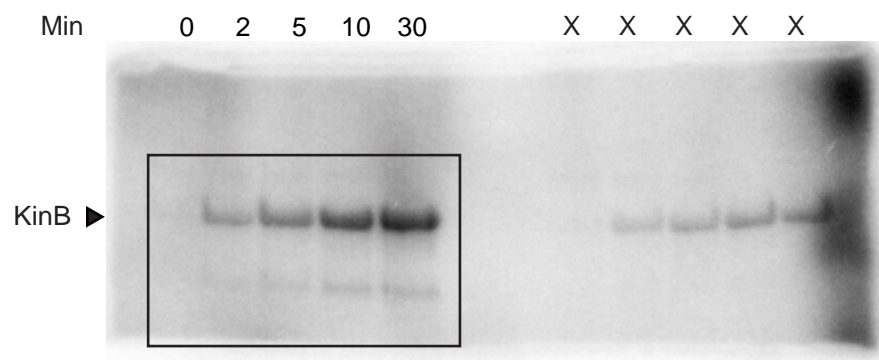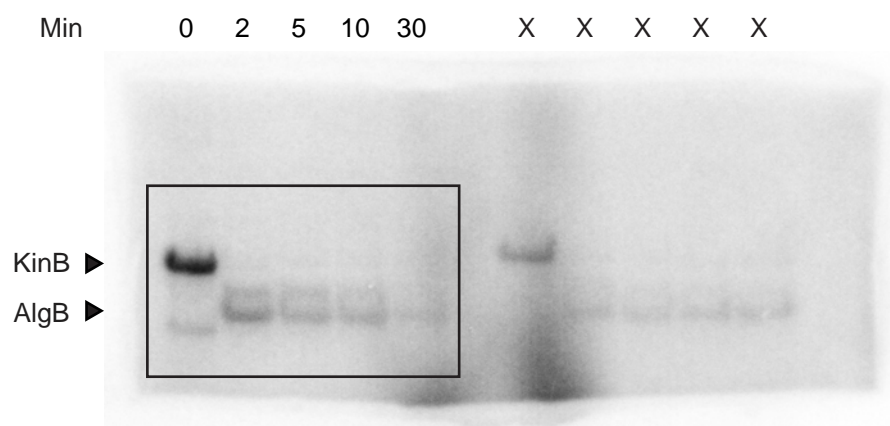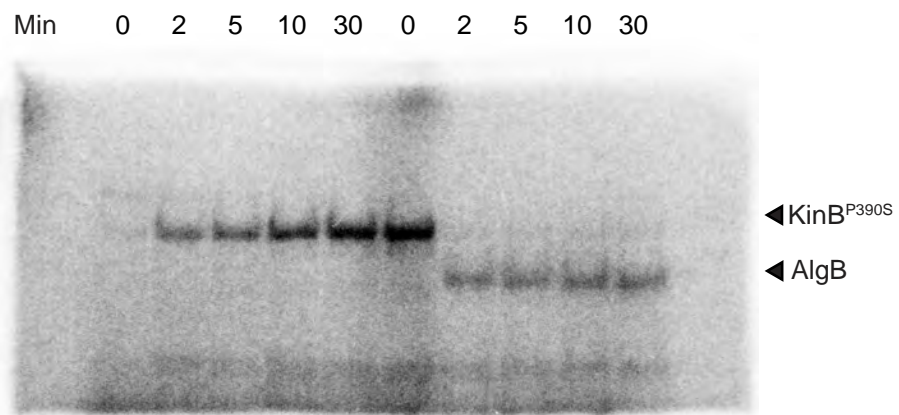

Supplement: S2 Data — (PDF) [file pbio.3000579.s013.pdf]
