# Supplementary material for: Photosensing and quorum sensing are integrated to control Pseudomonas aeruginosa collective behaviors
Source: PLoS Biol. 2019 Dec 12;17(12):e3000579. doi: 10.1371/journal.pbio.3000579 (PMC6932827; doi:10.1371/journal.pbio.3000579)
Supplement: S1 Table — (DOCX) [file pbio.3000579.s008.docx]

**S1 Table. Transposon insertion locations.**

| **PA14 ID^a^** | **PAO1 ID^a^** | **Gene name and description of encoded protien** |
| --- | --- | --- |
| PA14_19090 | PA3480 | *dcd*, deoxycytidine triphosphate deaminase |
| PA14_22060 | PA3241 | hypothetical protein |
| PA14_23420 | - | *zbdP,* zinc-binding dehydrogenase |
| PA14_23470 | PA3141 | *wbpM*, nucleotide sugar epimerase/dehydratase |
| PA14_24480 | PA3064 | *pelA*, extracellular polysaccharide biosynthesis protein |
| PA14_24490 | PA3063 | *pelB*, extracellular polysaccharide biosynthesis protein |
| PA14_32610 | PA2476 | *dsbG*, disulfide isomerase/thiol-disulfide oxidase |
| PA14_33270 | PA2411 | *pvdG*, pyoverdine synthetase |
| PA14_33700 | PA2396 | *pvdF*, pyoverdine synthetase |
| PA14_35720 | - | hypothetical protein |
| PA14_38510 | PA2009 | *hmgA*, homogentisate 1,2-dioxygenase |
| PA14_40860 | PA1830 | hypothetical protein |
| PA14_44070 | PA1580 | *gltA*, type II citrate synthase |
| PA14_52060 | PA0943 | hypothetical protein |
| PA14_58760 | - | *pilC*, type 4 fimbrial biogenesis protein |
| PA14_59630 | - | hypothetical protein |
| PA14_59800 | - | *pvrS*, two-component sensor kinase |
| PA14_66620 | PA5040 | *pilQ*, type 4 fimbrial biogenesis protein |
| PA14_66660 | PA5044 | *pilM*, type 4 fimbrial biogenesis protein |
| PA14_72390 | PA5484 | *kinB*, two-component sensor kinase |

a: annotation from www.pseudomonas.com [63]
