# Supplementary material for: Photosensing and quorum sensing are integrated to control Pseudomonas aeruginosa collective behaviors
Source: PLoS Biol. 2019 Dec 12;17(12):e3000579. doi: 10.1371/journal.pbio.3000579 (PMC6932827; doi:10.1371/journal.pbio.3000579)
Supplement: S2 Table — (DOCX) [file pbio.3000579.s009.docx]

**S2 Table. Suppressor mutations of the Δ*kinB* smooth colony biofilm phenotype.**

| **Suppressor** | **PA14 ID^a^** | **Gene name** | **Nucleotide position** | **Mutation** |
| --- | --- | --- | --- | --- |
| SM1045 | PA14_72380 | *algB* | 6447033 | Δ10 bp |
| SM1062 | PA14_72380 | *algB* | 6447033 | Δ10 bp |
| SM1063 | PA14_72380 | *algB* | 6447033 | Δ10 bp |
| SM1064 | PA14_72380 | *algB* | 6447033 | Δ10 bp |
| SM1067 | PA14_10700 | *bphP* | 919652 | Δ1603 bp |
| SM1068 | PA14_72380 | *algB* | 6447064 | Δ1 bp |
| SM1072 | PA14_72380 | *algB* | 6447033 | Δ10 bp |
| SM1073 | PA14_72380 | *algB* | 6447033 | Δ10 bp |
| SM1074 | PA14_10700 | *bphP* | 921631 | G 🡪 T |
| SM1149 | PA14_72380 | *algB* | 6446399 | Δ21 bp |
| SM1150 | PA14_10700 | *bphP* | 921151 | Δ12 bp |
| SM1151 | PA14_10700 | *bphP* | 921731 | G 🡪 T |

a: annotation from www.pseudomonas.com [63]
