# Supplementary material for: Photosensing and quorum sensing are integrated to control Pseudomonas aeruginosa collective behaviors
Source: PLoS Biol. 2019 Dec 12;17(12):e3000579. doi: 10.1371/journal.pbio.3000579 (PMC6932827; doi:10.1371/journal.pbio.3000579)
Supplement: S3 Table — (DOCX) [file pbio.3000579.s010.docx]

**S3 Table. Bacterial strains used in this study.**

| **Strain** | **Description** | **Reference** |
| --- | --- | --- |
| UCBPP-PA14 | Wild type *Pseudomonas aeruginosa* | Laboratory stock |
| SM32 | Δ*rhlR* | [21] |
| SM1040 | Δ*rhlR* Δ*kinB* | This study |
| SM1050 | Δ*kinB* | This study |
| SM1111 | pUCP18-*P_lac_-kinB* | This study |
| SM1112 | Δ*rhlR* Δ*kinB* pUCP18*-P_lac_-kinB* | This study |
| SM1116 | Δ*kinB* pUCP18*-P_lac_-kinB* | This study |
| SM1204 | *algB^STOP^* | This study |
| SM1212 | Δ*kinB algB^STOP^* | This study |
| SM1278 | Δ*kinB bphP^STOP^* | This study |
| SM1282 | *bphP^STOP^* | This study |
| SM1286 | pUCP18*-P_lac_-algB* | This study |
| SM1303 | Δ*rhlR* pUCP18*-P_lac_-algB* | This study |
| SM1326 | *bphP^STOP^* pUCP18-*P_lac_-algB* | This study |
| SM1377 | *3xFLAG-algB* | This study |
| SM1378 | Δ*kinB 3xFLAG-algB* | This study |
| SM1386 | Δ*kinB bphP^H513A^* | This study |
| SM1387 | *algB^STOP^* pUCP18*-P_lac_-algB* | This study |
| SM1388 | *bphP^H513A^* pUCP18-*P_lac_-algB* | This study |
| SM1413 | *algB^STOP^* pBBR1-MCS5*-P_lac_-3xFLAG-algB* | This study |
| SM1514 | *3xFLAG-algB kinB-SNAP* | This study |
| SM1523 | *3xFLAG-algB kinB^P390S^-SNAP* | This study |
| SM1535 | pBBR-MCS5*-P_lac_-bphP* | This study |
| SM1543 | *algB^STOP^* pBBR1-MCS5-*P_lac_-bphP* | This study |
| SM1562 | *algB^STOP^* pBBR1-MCS5-*P_lac_-3xFLAG-algB^D59N^* | This study |
| SM1563 | *algB^STOP^* pUCP18-*P_lac_-algB^D59N^* | This study |
| SM1597 | *algB^D59N^* | This study |
| SM1598 | Δ*kinB algB^D59N^* | This study |
| SM1617 | *3xFLAG-algB bphP-3xFLAG* | This study |
| SM1618 | *3xFLAG-algB bphP^H513A^-3xFLAG* | This study |
