# Supplementary material for: Photosensing and quorum sensing are integrated to control Pseudomonas aeruginosa collective behaviors
Source: PLoS Biol. 2019 Dec 12;17(12):e3000579. doi: 10.1371/journal.pbio.3000579 (PMC6932827; doi:10.1371/journal.pbio.3000579)
Supplement: S4 Table — (DOCX) [file pbio.3000579.s011.docx]

**S4 Table. Plasmids used in this study.**

| **Plasmid** | **Description** | **Reference** |
| --- | --- | --- |
| pEXG2 | Allelic exchange vector with pBR origin, gentamicin resistance, *sacB* | [62] |
| pUCP18 | E. coli-Pseudomonas Amp^r^ shuttle vector | Laboratory stock |
| pBBR1-MCS5 | E. coli-Pseudomonas Gent^r^ shuttle vector | Laboratory stock |
| pIT2 | ISlacZ/hah transposon mutagenesis vector | [25] |
| pET21b | Protein expression vector, Amp^r^ | Laboratory stock |
| pET28b | Protein expression vector, Kan^r^ | Laboratory stock |
| pSP201 | pET21b-*bphP-His6* | This study |
| pSP202 | pET21b*-kinB-His6* | This study |
| pSP203 | pET28b*-His6-algB* | This study |
| pSP204 | pET21b*-bphP^H513A^-His6* | This study |
| pSP205 | pET28b*-His6-algBPpu* | This study |
| pSP206 | pET28b*-His6-algB^D59N^* | This study |
| pSP207 | pET21b*-kinB^P390S^-His6* | This study |
| pSP208 | pET28b*-His6-ntrC* | This study |
| pSP209 | pET28b*-His6-algBRce* | This study |
| pSP210 | pET28b*-His6-algBAxy* | This study |
