# Supplementary material for: Photosensing and quorum sensing are integrated to control Pseudomonas aeruginosa collective behaviors
Source: PLoS Biol. 2019 Dec 12;17(12):e3000579. doi: 10.1371/journal.pbio.3000579 (PMC6932827; doi:10.1371/journal.pbio.3000579)
Supplement: Attachment — Submitted filename: Mukherjee Response to reviewers.docx [file pbio.3000579.s014.docx]

Dear Editor,

Thank you for handling our manuscript. We are grateful for such overwhelmingly positive reviews, with referees using terms including “masterful”, “amazing”, “a real tour de force!”, “well done”, “of interest to a broad range of scientists”, “nice data” and “effective and convincing”. We are also grateful to have their good suggestions to help us improve the work. We have made all of the requested text changes. We engineered the requested strain with the *algB^D59N^* mutation on the chromosome and provided the data for its phenotype. We added new data showing that far-red light represses solid surface associated (SSA) biofilm formation in WT *P. aeruginosa*. We also used quantitative RT-PCR to demonstrate that introducing a stop codon into *algB* does not alter *kinB* expression. Below we provide our point-by-point responses to your comments and to those of the referees. We have also included our response to the Academic Editor’s note. Editor/Referee text is in black and our responses are in red text. We hope you now find that our manuscript meets the standards of *PLOS Biology*.

Thank you for your consideration.

Bonnie Bassler, on behalf of all authors.

**Editor Comments:**

You'll see that while the reviewers' assessments of your study are broadly positive, each of them raises some concerns that will need addressing, some with additional experimental data. IMPORTANT: The Academic Editor notes that some of the papers mentioned by reviewer #2 do reduce the novelty of your claims somewhat, increasing the importance of demonstrating the translational potential of your findings more directly, as requested by reviewer #4, who recommends that you assess the effect of light on a mouse burn wound model.

We very much appreciate the remarkably positive referee comments about the rigor, depth, and scope of the work in our manuscript. Respectfully, however, we disagree that the articles mentioned by Reviewer #2 reduce the novelty of our work. Those studies could not have led us to the RhlR-AlgB-KinB-BphP circuitry nor to the discovery of photo sensing in *P. aeruginosa* that we report here by using forward genetic screens.

Specifically, Schuster et al. 2003 performed transcriptome analysis and identified 638 genes that are quorum-sensing regulated, one of which is *bphP*. Schuster did not study BphP function or its regulation. *bphP* is simply one gene in a list of 600 other genes.

With regard to Barkovits et al. 2011, that work showed that LasR activates *bphP* expression. This same study concluded that *P. aeruginosa* does not sense nor respond to light, and the authors wrote that BphP detects an unknown signal and must convey information to an unknown response regulator. Those results neither track with nor inform our studies. We prove that BphP does indeed detect light, that its response regulator is AlgB, and the phenotypes controlled are biofilm formation and virulence. Moreover, we show that KinB antagonizes BphP.

With regard to Chand et al. 2011, respectfully, there is a confusion. Deborah Hung’s group worked on KinB. They did not know about BphP. In contrast to the reviewer’s recollection, the Hung group showed that the Δ*kinB* mutant is indeed attenuated for virulence in zebrafish. In fact, the Chand et al. paper concluded that KinB functions independently of its response regulator AlgB. Again, these findings have no bearing on ours and they could not have led us to the discoveries that we report in our manuscript.

We are grateful for the Academic Editor’s response to our email discussing the Chand et al. work and whether, given all the new data we provide, inclusion of animal experiments should fall within the scope of this manuscript.

Regarding Chand et al., the Academic Editor wrote, “I’ve now spent some time reading the Bassler responses *and* some of the relevant literature. It does seem like there’s a mistake in reviewer #2’s point #3 - when s/he says BphP was already shown to play a role in infection, I think s/he is mistaken and means KinB. However, in the vein of rev #2’s comments: I think they should be encouraged to better clarify in their Introduction exactly what was already known. For example, the Hung work on KinB (ref 24) seems central to this paper, but is never cited or adequately discussed in the Introduction.”

We thank the Academic Editor for his/her insight. We certainly want to be sure that we clearly lay out the important findings on KinB that have come before ours. In response to the Academic Editor’s comment, we have expanded our description of the Chand et al. work on KinB in the revised introduction. We write, “KinB and AlgB are a two-component system (TCS) sensor HK and partner RR, respectively [21,27]. KinB is required for acute infection in zebrafish embryos where it controls production of virulence factors such as pyocyanin and it promotes motility [27,28].”

Regarding performing animal studies, the Academic Editor wrote, “Bassler et al do note in the Discussion that a role for BphP was apparently already suggested for P. syringae, but it has not apparently been linked to pathogenesis for P. aeruginosa. And it certainly hasn’t been shown to regulate AlgB as shown in this manuscript. I think given this plus the positivity of the reviewers overall, I’m inclined at this point to let go the demonstration of how light impacts P. aeruginosa in a mouse model.”

We thank the Academic Editor for suggesting that testing the effects of light on *P. aeruginosa* virulence in animals can become a future phase of this work. We absolutely agree with Referee 4 that animal work is an exciting and worthy direction to take.

We hope the referees will also agree that the novelty of our work arises from our demonstration that the non-photosynthetic global pathogen *P. aeruginosa* alters virulence and biofilm behavior in response to light, that we have identified the complete photo sensing signaling cascade in *P. aeruginosa*, and that we have solved the long standing mystery regarding the identity of the response regulator for the BphP orphan kinase. Our work provides the blueprint for investigations of photo regulation of bacterial physiology in *P. aeruginosa* as well as members of diverse bacterial phyla that are non-photosynthetic.

*****************************************************

REVIEWERS' COMMENTS:

Reviewer #1:

General Comments

In a masterful random transposon search, these investigators discovered that the transmembrane histidine kinase, KinB, which is in an operon with an alginate 2-component regulator, AlgB, affects biofilm formation in Pseudomonas aeruginosa. Their findings show that KinB is a dual kinase/phosphatase, but mostly a phosphatase during their in vivo conditions. Through another mutant search, they find that BphP-AlgB-KinB forms a “three-component” system where AlgB is activated by the kinase activity of light-sensor BphP and inhibited by the phosphatase activity of KinB. Furthermore, they demonstrate that P. aeruginosa biofilm formation can be modulated simply by tuning the intensity of far-red light in which the strain is grown. This is amazing that Pseudomonas virulence phenotypes are under photo control, and that the molecular genetic mechanisms can be explained.

We thank Reviewer #1 for such generous appreciation of our work.

Specific Comments

1. Line 107 says WT PA14 exhibits a rugose-center/smooth-periphery colony biofilm, and the ∆rhlR mutant forms a larger hyper-rugose biofilm. Line 109 says RhlR impedes biofilm formation, and yet the wild-type (RhlR+) is said to make a “biofilm.” This is confusing, so a better definition of biofilm is needed instead of calling everything a biofilm. By definition, does WT PA14 (RhlR+) form a biofilm even though it is mostly smooth? There appears to be 3 phenotypes on the Fig 2A plates: WT semi rugose biofilm, hyper-rugose biofilm and smooth biofilm. Line 123 says kinB “mutants failed to form biofilms…” Should this say “mutants formed smooth biofilms” to be consistent? Should line 128 say “KinB is essential for ‘hyper-rugose’ biofilm formation”? Why is smooth less of a biofilm? A short description of how the Congo red agar phenotypes compare with other types of Pa biofilms (e.g., static ring, flow cell) would be helpful.

The colony biofilm assay used in our work is identical to the assay we published previously in 2017 and 2018 manuscripts (Mukherjee et al., PLOS Pathogens, Mukherjee et al., PNAS), and this assay is routinely used by other research groups. The accepted term for these communities is “colony biofilms” and we have used that term throughout this work. *P. aeruginosa* PA14 requires Pel, the primary biofilm matrix exopolysaccharide to form the characteristic rugose-center/smooth-periphery colony biofilm. A Δ*kinB* mutant shows a severe decrease in *pelA* expression (Fig 2B), fails to produce Pel, does not form colony biofilms, and therefore appears smooth in our assay. Thus, we conclude that KinB is essential for colony biofilm formation.

As requested, we have clarified the known role of Pel in *P. aeruginosa* PA14 colony biofilm formation under different conditions in the legend to Fig 2. We have added the following sentence: “The *pelABCDEFG* operon encodes proteins required for the production of the Pel polysaccharide, which is essential for the formation of pellicles, solid surface-associated biofilms, and colony biofilms [29].”

In addition, to further address the reviewer’s question, we have now assessed the effect of light on the formation of solid surface associated (SSA or static ring) biofilms by *P. aeruginosa* PA14. We find that, consistent with what we show for colony biofilms, far-red light represses SSA biofilm formation in the WT. The Δ*kinB* mutant shows a severe decrease in SSA biofilm formation under ambient and far-red light but produces SSA biofilms in the dark. Finally, light-driven repression of SSA biofilm formation requires the presence of BphP and AlgB. We have added these data as new Fig S6B and the companion text are provided in the results section.

2. Line 117 – (Fazli et al., 2014) was not added to the References list and should have a # in the text.

We thank the reviewer for this comment and the good eye! We have corrected the citation.

3. Line 139 says KinB is a HK that transfers phosphate to AlgB (ref. 19), but the Fig. 1 model contradicts this and shows KinB + ?stimulus as a phosphatase that only dephosphorylates AlgB. This is confusing, so maybe the legend should state it also phosphorylates AlgB in the presence of an unknown KinB stimulus.

As requested, we have changed Fig 1 and updated the legend to clarify the dual role of KinB.

4. Fig. 2A has a lot of genotypes/phenotypes to sort through. The conclusion at Line 147 says AlgB represses (WT, semi-rugose) biofilm development. But WT PA14 is algB+ and has a WT semi-rugose biofilm phenotype [as did an algB(stop) mutant, which should be smooth according to line 147]. The kinB mutant has a smooth biofilm, so it’s fair to say KinB activates the WT biofilm phenotype. It is appropriate to look at AlgB, the logical partner of KinB, and a kinB algB mutant goes back to a WT phenotype; that fits. I’m not sure that algB overexpression, producing a smooth phenotype (Fig 2A-12) is a reliable phenotype. Perhaps Line 147 should say that “these data suggest that …”

As recommended, we have changed the sentence to read, “Thus, these data suggest that KinB activates while AlgB represses biofilm development.”

5. Line 160 invokes an unknown negative regulator of biofilms (controlled by AlgB) based on strains with algB overexpressed vs. algB(D59N) unphosphorylated. In the WT (Fig 2A-1), the strain is chromosomally AlgB+ but shows the WT semi-rugose biofilm. Perhaps WT with algB overexpressed (Fig 2A-12) has too much AlgB~P, causing an artifact? It may require having algB(D59N) in single copy in the chromosome to really confirm the conclusions.

When *algB* is overexpressed in our system, it enables the cells to override the KinB phosphatase function and, in turn, repress colony biofilm formation. Moreover, *algB* overexpression has no effect on colony biofilm formation when the BphP kinase is absent (Fig 3C and 3D), eliminating concerns that the phenotypes we are assaying stem from artifacts of overexpression.

Nonetheless, to ensure that we are convincing and to address the reviewer’s concern, we have included the colony biofilm phenotype of the *P. aeruginosa* mutant carrying *algB^D59N^* on the chromosome as new supplemental Fig S3B. The new data show that, identical to the result from the plasmid-based experiment, *P. aeruginosa* carrying *algB^D59N^* on the chromosome in the WT and in the Δ*kinB* mutant both produce colony biofilms. These results show that AlgB fails to function when aspartate residue 59 cannot be phosphorylated.

We presume that AlgB~P represses colony biofilm formation indirectly by activating the expression of a gene encoding another repressor. We say this because AlgB is an NtrC-type response regulator and as far as is known, this family of response regulators function exclusively as transcriptional activators.

6. Line 170. This is amazing that AlgB~P is detected in a kinB mutant, but not in WT, suggesting (line 180) that some His-kinase other than KinB phosphorylates AlgB!

7. The discovery of the bphP gene as encoding the kinase of AlgB was excellent genetics. Much of the manuscript is on the effect of light on phenotypes, which is well done. Perhaps the lesson is that we can’t incubate our plates in just dark incubators anymore as this may mask other phenotypes.

We appreciate that (comments 6 and 7) the reviewer shares our excitement about photo regulation of bacterial behaviors. It should be fascinating to rigorously test common phenotypes, and screen for new ones, when light levels are precisely controlled.

Reviewer #2:

This manuscript by Mukherjee and colleagues describes what they term a “three component system” that is comprised of the known P. aeruginosa regulators, KinB, AlgB, and BphP. The authors provide genetic and biochemical evidence that the KinB kinase dephosphorylates AlgB while the bacteriophytochome kinase, BphP, phosphorylates AlgB. These three proteins coordinately control biofilm formation and the expression of virulence genes. The discoveries outlined in the manuscript provide new understanding of genes that function downstream of the quorum regulator RhlR to control virulence and biofilm gene expression. Moreover, the authors uncover a role for light in the regulation of these genes through the known red-light sensor, BphP.

This study will be of interest to a broad range of scientists, particularly investigators studying bacterial pathogenesis and photobiology. The study is well written and the data are easy to follow.

We thank Reviewer #2 for the enthusiastic endorsement of our work and for saying that we provide “new understanding”. That was our goal.

The authors should consider the suggestions below before publication.

1)	BphP has been previously described as a quorum regulated gene by Schuster et al (J. Bact 2003, 185:2066) and by Barkovits et al (Microbiology 2011, <https://doi.org/10.1099/mic.0.049007-0>) so there was some indication that this gene would play a role in some quorum pathway. It’s likely worth touching on this in either the introduction or discussion.

As outlined above, these earlier studies, while informative, could not have led us to our conclusions nor to the RhlR-AlgB-KinB-BphP circuitry that we discover and report here by using forward genetic screens.

Specifically, Schuster et al. 2003 performed transcriptome analysis and identified 638 genes that are quorum-sensing regulated, one of which is *bphP*. Schuster did not study BphP function or its regulation. *bphP* is simply one gene in a list of 600 other genes. We show that it is RhlR that controls *algB*, that AlgB requires phosphorylation by BphP to function, that BphP detects light to control virulence and biofilms, and that BphP requires AlgB to do so.

With regard to Barkovits et al., that work showed that LasR activates *bphP* expression. That same study concluded that *P. aeruginosa* does not sense nor respond to light, and the authors wrote that BphP detects an unknown signal and must convey information to an unknown response regulator. Those results neither track with nor inform our studies. We prove that BphP does indeed detect light, that its response regulator is AlgB, and the phenotypes controlled are biofilm formation and virulence. Moreover, we show that KinB antagonizes BphP.

We have focused on the Rhl quorum-sensing system in this manuscript. Based on the findings here, we have launched a new study of the individual and combined roles of the Las and Rhl quorum-sensing systems on photo sensing.

2)	Along this same line, BphP has also been shown to be regulated by RpoS (Barkovits 2011). Therefore, it is likely that multiple pathways impinge on the BphP-AlgB axis. This is likely a minor discussion point should the authors choose to address it.

We thank the reviewer for this idea. We have added a sentence about Las and RpoS control of *bphP* expression to the discussion and we added Barkovits et al. to our citation list. The new sentence reads, “We note that *bphP* expression has been reported to be controlled by LasR/I quorum sensing and the stationary phase alternative sigma factor RpoS [37] suggesting that other sensory cues can be integrated into the AlgB-KinB-BphP circuit.”

3)	The idea that light, via BphP, may be playing a role in infection/parthogenesis is interesting. However, the Hung group has shown that a BphP mutant is not attenuated in a vertebrate model (Chand et al, J. Bact 2011). Clearly, there is more to do on the infection with respect to light and other treatments, but this result from the Hung group could be mentioned/discussed.

Respectfully, there is confusion on this point. Deborah Hung’s group worked on KinB. They did not know about BphP. In contrast to the reviewer’s recollection, the Hung group showed that the Δ*kinB* mutant is attenuated for virulence in zebrafish. Based on our results, we would predict that the Δ*kinB* mutant should display an attenuated virulence phenotype because in the absence of KinB, BphP phosphorylates AlgB, and AlgB~P represses virulence factor production.

To ensure that we clearly lay out the important findings about KinB that came before ours, in the revised Introduction, we better explain the results of Chand et al. regarding KinB. We write “KinB and AlgB are a two-component system (TCS) sensor HK and partner RR, respectively [21,27]. KinB is required for acute infection in zebrafish embryos where it controls production of virulence factors such as pyocyanin and it promotes motility [27,28].”

4)	I may have missed it, but does apo BphP have the same activity and response regulator specificity?

We do not know the answer to this question. As reported by Bhoo et al., Nature. 2001;414: 776–779, BphP requires the biliverdin cofactor for autophosphorylation *in vitro*. Based on this referee question, we have now also included data showing that apo-BphP does not autophosphorylate *in vitro* (Fig S4A). Because apo-BphP is not phosphorylated, we cannot test specificity of phosphotransfer to AlgB.

5)	It is clear from the authors’ data that red-light has the most potent effect of BphP-dependent regulation of gene expression. However, the result that blue, red, and far-red light all activate BphP goes against what I understand about the energy dependence of the bacteriophytochromes. This is a very interesting piece of data that is worth discussing more. In particular, I think it would be useful to revisit some the bacteriophytochome literature to see if there are other examples of these proteins that are activated by blue light. Is there any indication from the published Pa-BphP absorption spectra that this protein can be activated by blue light?

We agree with the reviewer that BphP detection of blue, red, and far-red light is indeed interesting. As far as we understand, bacteriophytochromes are reversible photo-switches that are activated as kinases either by red or far-red light, but not both. There are reports of algal phytochromes that have blue-shifted wavelength preferences, but again, they are thought to function as photo-switches. The available PaBphP absorption spectrum shows a minor peak at 420 nm (blue) in addition to peaks at 690 nm (red) and 750 nm (far-red) but the spectrum does not offer mechanistic insight into activation of BphP kinase activity by all three light wavelengths. In ongoing biochemical and genetic studies, we are examining the native BphP spectral properties, making mutants with altered light detection phenotypes, and defining the requirements for detection of different wavelengths of light.

As requested, we have added a paragraph in the discussion to address BphP promiscuity in photoreception. The new paragraph reads, “Bacteriophytochromes are thought to function as reversible photo-switches that convert between red-absorbing and far-red-absorbing states [17]. The *P. aeruginosa* BphP kinase has been reported to be activated by far-red light *in vitro* [22]. Interestingly, however, the published biliverdin absorption spectrum possesses peaks at blue, red, and far-red light [53] hinting at promiscuity in light detection. Based on our *in vitro* and *in vivo* data, we suggest that *P. aeruginosa* BphP is active as a kinase in both the red and far-red states as well as under blue light. We are currently investigating the mechanism by which the BphP-BV holo-complex is activated by different wavelengths of light.”

6)	A handful of references (e.g. 23 & 29, others) are missing the journal name.

We thank the reviewer for his/her keen reading. We regret the omissions. We have corrected the references.

Reviewer #3:

[identifies himself as Carl E Bauer]

This manuscript from the Bassler laboratory effectively and convincingly demonstrates that P. aeruginosa has a very interesting complex phosphoryl relay that controls both biofilm formation and virulence factor production in response to light exposure. Their study identifies all of the major components of this signaling event and uses a strong and convincing combination of in vivo genetics and in vitro biochemistry to show roles of these various signaling components. My overall conclusion is that this manuscript is a real tour de force! It’s a stunningly good example of a complete body of work that will likely open up a whole new avenue of research on the role of photoreceptors in the control of biofilm formation and virulence.

We thank Reviewer #3 for being so excited about our new work. We especially appreciate the term “tour de force” with an exclamation point. It is not often one gets to read referee comments like that, and it reminds us how much fun it is to do what we do!

I only have a few minor suggestions.

Line 67-69: B12 is also recently recognized as a light absorbing chromophore for a photoreceptor that controls photosynthesis gene expression. B12 thus represents a new member of the photoreceptor family. See: Trends in Biochemical Sciences. 41:647-50; Elife. 2018 Oct 3;7. pii: e39028. doi: 10.7554/eLife.39028.

As recommended, we have added the B12 photoreceptor to the classes of photoreceptors named in the introduction. We have also added the suggested citations. They are reference numbers #14 and #15 in the updated citation list.

The new introductory sentence reads, “Photoreceptors fall into seven families depending on the structure of the light-absorbing chromophore: rhodopsins, xanthopsins, cryptochromes, LOV domain-containing phototropins, blue-light sensing using flavin (BLUF)-domain proteins, cobalamin (Vitamin B12)-binding proteins, and phytochromes [12-15].”

Figure 1: Is there any evidence that BphB is membrane associated? As far as I know, bacterial phytochromes are not membrane bound. Also, this figure may be oversimplified. As shown in lines 271-273, KinB is capable of autophosphorylation and transferring a phosphate to AlgB in vitro. The authors rightfully conclude that kinB may be providing a phosphate to AlgB in vivo but the conditions for this need to be worked out. The likely dual role of KinB phosphorylation and dephosphorylation of AlgB is not reflected by this figure.

We thank the reviewer for this good suggestion. *P. aeruginosa* bioinformatic analyses suggest that BphP is localized to the cytoplasmic membrane (PSORTb 3.0 analysis), which is why we had depicted PaBphP as membrane anchored. However, we cannot find any experimental evidence to support this localization in the literature. Therefore, as recommended by the expert referee, we have redrawn Fig 1 with BphP in the cytoplasm. We have also changed the figure to show the dual role of KinB, as suggested.

Reviewer #4:

In this manuscript, the authors demonstrate that BphP is a SK that can phosphorylate the RR AlgB. AlgB has been known for some time to work with its cotranscribed SK KinB, however KinB appears to function primarily as a phosphatase rather than a kinase. This manuscript solves the puzzle of what else might be phosphorylating AlgB, by identifying the far red light sensing SK BphP.Following phosphorylation assays, the methods employed include basic in vitro assays of phenotype and biofilm formation. The manuscript is convincing as far as it goes, although for a journal like PLoS Biology I would expect to see more of the medical relevance of BphP’s role explored e.g. employing an animal infection model to assess virulence of BphP mutants vs WT, and also testing the role of light as a therapy.

We thank Reviewer #4 for critical reading of our manuscript. Respectfully, we disagree that publication in PLOS Biology requires medical relevance. Our understanding is that PLOS Biology publishes studies that are discovery-driven and that appeal to a broad audience. From the webpage of PLOS Biology: “PLOS Biology features works of exceptional significance, originality, and relevance in all areas of biological science, from molecules to ecosystems…”. We take the superlatives the referees wrote, “masterful”, “amazing”, “a real tour de force!”, “well done”, “of interest to a broad range of scientists”, “nice data” and “effective and convincing” to indicate that, with the revisions we provide, we have met the criteria for publication.

In the manuscript, we stated that the work presented sets the stage for animal studies so we are aware that, in the future, medical relevance should be tested. We hope the referee can understand that for us to set up a mouse colony requires a wholly new focus for our lab along with significant new funding to finance such an endeavor. It would take a year or years to establish a mouse colony and acquire approval for new light-based treatments of infection and then to perform them. To us, such a request seems beyond the scope of this initial paper reporting the discovery of a new signal transduction circuit and its target genes.

Had we submitted this work to PLOS Pathogens, medical relevance would be key, but our goal was to focus on the biology of the system we discovered, hence our choice of a PLOS Biology submission. We hope the referee can agree.

Other comments:

Line 38 and elsewhere: “three-component” system – please rephrase. This is still a two-component system (i.e. a system with sensor kinase(s) and response regulator(s)). This two-component system happens to have two sensor kinases (KinB and BphP) and one response regulator (AlgB). One of those SKs is primarily a phosphatase (KinB) while the other is primarily a kinase (BphP).

As requested, we have altered the text. The new sentence reads, “We propose that in the KinB-AlgB-BphP system, AlgB functions as the node at which varied sensory information is integrated.” We have also removed the phrase “three-component system” from the remainder of our manuscript.

Line 39: “We propose that KinB-AlgB-BphP constitutes a “three-component” system with AlgB acting as the node at which varied sensory information is integrated. This network architecture provides a mechanism enabling the bacteria to integrate at least two different sensory inputs, quorum sensing and light, into the control of collective behaviors”. Would be helpful to clarify that the link to quorum sensing is coming in simply at the transcriptional level (i.e. RhlR activating transcription of AlgB and KinB) rather than as a consequence of AlgB being controlled by the sensor kinases KinB & BphP.

As requested, we have altered the text. The new sentence reads, “This network architecture provides a mechanism enabling the bacteria to integrate at least two different sensory inputs, quorum sensing (via RhlR driven activation of *algB*) and light (via BphP-AlgB), into the control of collective behaviors.”

Line 87: “We demonstrate that P. aeruginosa biofilm formation and virulence gene expression are repressed by far-red light”. I am puzzled as to why no images/data are shown for the single bphPstop mutant in figure 4A, C & D. Could these be added? These would show that it was bphP that was acting as the far-red light sensor. To some extent this information is in Figure 5, but it would be neater to have in figure 4 too.

The original Fig 4 is a full-page figure. Due to space constraints, we chose to show the colony biofilm data for the Δ*kinB algB^STOP^* and Δ*kinB bphP^STOP^* double mutants in the main figure. Our logic was that when either AlgB or BphP is eliminated from the Δ*kinB* mutant, light-mediated repression of biofilm formation is relieved under every light condition tested.

As requested, in the revised work, we have added the biofilm data for the *bphP^STOP^* and *algB^STOP^* mutants in both the WT and the Δ*kinB* parent strains by making a new supplemental figure, Fig S6A.

Table S1: To help the reader please add PAO1 gene names to this table.

As requested, we have added *P. aeruginosa* PAO1 gene names to Table S1.

Line 139-147. The AlgBSTOP mutant is an unusual choice of mutant. AlgB and KinB are encoded together in an operon, with algB being the upstream gene; there is a danger that a premature stop codon within algB would significant impact kinB expression.

To address the reviewer’s concern, we have added data as Fig S1B showing that *kinB* expression is unchanged between the WT and the *algB^STOP^* mutant.

Line 154: Assumption that D59N mutant of algB is not phosphorylatable. Cite Fig S4 here as this isn’t always the case eg. E. coli CheYD57N is phosphorylated at serine 56 instead (Appleby and Bourret, 1999).

As requested, we have cited Fig S4A here.

Figure S3: Legend mentions panels A-E but only A-C are present.

We thank the reviewer for this comment and his/her good eye. We regret that the panels for Fig S3D and S3E were omitted. We have added those data back to the revised manuscript.

In vitro phosphorylation assays: Nice data. Line 614 - Please specify the actual protein concentrations used for these (it is not clear enough to say equimolar).

As requested, we have now included the protein concentrations to the Methods section. For the referee’s reference, 5 µM each of AlgB, BphP, and KinB were assayed.

Figure 3 and elsewhere: Cognate needs to be defined carefully and explicitly. There are two different versions of it in the literature: i) it can be used to mean transcribed from the same place e.g. within an operon or ii) the SK that recognises and phosphorylates the RR.

As requested, we have changed “cognate” to “partner” throughout the manuscript.

Figure 3: Agreed that BphP is “a” cognate HK for AlgB but not convinced that it is “the” (as in the only) HK for AlgB. What about KinB? Could there be others?

We meant that BphP is the partner HK for AlgB under the conditions of our experiments. To address this comment, we have changed the title of Fig 3 to “BphP and AlgB are a two-component sensor kinase-response regulator pair”.

Line467 onwards: Too speculative. How much light would P. aeruginosa be exposed to when infecting humans/mammals? Inside the lungs is pretty dark whether it is day or night. Medical significance is unclear.

We agree with the referee that inside the lungs it is dark. Therefore, as our work suggests, the lack of light would drive *P. aeruginosa* to form biofilms and produce virulence factors because light-dependent BphP-AlgB-mediated repression of biofilms and virulence would not occur in lung tissue. We have clarified our thinking in the discussion so readers can understand that darkness promotes virulence and biofilm formation.

Line486: The idea of testing the effect of light on a burn wound infection is exciting and would massively increase the impact of this paper (if it works). I recommend the authors do these experiments – there are plenty of good mouse burn wound models available.

As mentioned above, we hope we have already met the criteria for publication in PLOS Biology as stated on the journal’s home page. We agree that animal studies are exciting future experiments, and we state exactly that in the manuscript. However, we are a bacterial genetics/biochemistry lab that does basic science. We do not currently have the financial resources nor the facilities to undertake such experiments. We hope the referee and the Academic Editor can see that launching animal studies is beyond the scope of this paper.
